# Surgical Intervention vs. Radiation Therapy: The Shifting Paradigm in Treating Metastatic Spinal Disease

**DOI:** 10.7759/cureus.3406

**Published:** 2018-10-03

**Authors:** Robert Le, Jeremy D Tran, Mel Lizaso, Ramin Beheshti, Austin Moats

**Affiliations:** 1 Internal Medicine, University of Central Florida College of Medicine, Orlando, USA; 2 Orthopaedics, University of Central Florida College of Medicine, Orlando, USA; 3 Radiation Oncology, University of Central Florida College of Medicine, Orlando, USA; 4 Oncology, University of Central Florida College of Medicine, Orlando, USA; 5 Radiology, Yale School of Medicine/Yale New Haven Health, New Haven, USA

**Keywords:** spinal surgery, radiation therapy, metastatic spinal disease, stereotactic body radiotherapy

## Abstract

The spine is one of the most common sites to which metastatic cancer is likely to spread and is one of the leading causes of morbidity and mortality in cancer patients. While no medical treatments have been definitively shown to extend the life expectancy of patients with spinal metastasis, interventional options may be the only viable option in improving outcomes. Currently, two main options exist: surgical resection and radiotherapy, with radiotherapy being the primary treatment modality. In this review, we discuss the research comparing the efficacy and outcomes of radiotherapy and surgical resection in treating spinal metastasis. We conclude that while radiosurgery will continue to remain a major treatment modality, surgical intervention has proven to have equal to or superior outcomes at improving function, symptoms, and life expectancy for patients with metastatic spinal disease and should be considered a primary modality in an expanding subset of patients.

## Introduction and background

The spine is the third most common site to which metastatic cancer is likely to spread, following the lungs and liver. However, it is the most common osseous site of metastatic invasion, comprising 70% of all metastatic osseous sites. The most common primary sites of these metastatic cancers are the lung (31%), breast (24%), gastrointestinal (GI) tract (9%), and prostate (8%). It is estimated that 5 - 30% of all patients with metastatic cancer will have vertebral involvement. The average length of survival of these patients is 10 months, reflecting the aggressive nature of the malignancy [1].

In addition, spine metastasis is one of the leading causes of morbidities in cancer patients. The symptoms and clinical course of these patients who experience metastases are similar, regardless of the primary neoplastic histology. The most common, and often initial, symptom is chronic back pain that is frequently worse at night and afflicts approximately 90% of patients [1]. The weakened bone can also result in compression fractures and spinal instability, presenting as sudden severe pain that is exacerbated by movement and axial load. Further disease progression can lead to neurological deficits, resulting in radiculopathy from nerve root compression or myelopathy from spinal cord compression. Radiculopathy presents as upper or lower extremity pain, weakness, and numbness, depending on whether the cervical or lumbar nerve roots are being compressed. Myelopathy presents with worsened weakness and sensory loss, paralysis, and autonomic dysfunction, including bowel and bladder incontinence. It is estimated that 21 - 48% of patients with myelopathy will become paraplegic over the course of the disease. In addition, myelopathic symptoms are a poor prognosticator, indicating an estimated survival of approximately three to seven months [2].

Currently, medical treatments only provide symptomatic relief with no significant improvement in clinical outcomes or life expectancy. NSAIDs, steroids, and opioids are commonly used to treat the musculoskeletal pain, while antiepileptic drugs, such as gabapentin, are used to treat the neuropathic pain. However, the only two viable options with improvement in outcomes for patients with spinal metastasis are radiation and surgical therapy. Since the 1980s, radiotherapy remains the primary modality, while surgery is only reserved for cases of radiation resistance, spinal cord compression, and spinal instability [3]. However, recent literature and improved surgical techniques have challenged this paradigm, suggesting an expanded role for surgical intervention. The objective of this paper is to characterize and compare the outcomes of radiation versus surgical intervention in treating spinal metastasis at improving function, alleviating symptoms, and prolonging life expectancy.

## Review

Stereotactic radiosurgery versus radiotherapy

Hashmi et al. conducted a multi-institutional pooled analysis of metastasis to the spine in patients with conventional external beam radiation therapy followed by re-irradiation stereotactic body radiation on the spine. Two hundred and fifteen cases were reviewed, with 247 spinal target volumes treated within seven settings/institutions that were part of the Elekta Spine Study Consortium (ESSC). These institutions were similar in terms of technique and equipment. Survival was dependent and calculated within a patient basis, and the control was calculated based on volumes treated. Both measurements were based on the Kaplan-Meier method. Local control was defined as imaging-based progression within the stereotactic body radiation therapy (SBRT) target volume. Sixty percent of the spinal target volumes were treated with single fraction SBRT and the results indicated a median of 16.6 grays (Gy) and 40% with multiple-fraction SBRT resulted with a median of 24 Gy. Equivalent dose in a 20 Gy fraction was calculated for the SBRT. The median time from conventional external beam radiation and re-irradiation stereotactic body radiation was 13.5 months and the median duration of the patient follow-up was 8.1 months. Based on the study, re-irradiation with stereotactic body radiation following radiation beam therapy is effective in yielding safe treatment but a randomized trial would be required to determine effectiveness [4].

A review by Gagnon et al. looked at randomized double-blinded trial studies in relation to the effects of treatment options, including differences between radiotherapy and radiosurgery for patients with metastatic bone disease. The patients were from Georgetown Hospital between the years of 2002 - 2003. Safety, pain, and quality of life following radiosurgical and radiation therapy were assessed and reviewed. The review included a prospective trial which observed 200 randomized patients with spinal tumors. The patients were treated through multisession stereotactic radiosurgery versus standard radiotherapy. Groups were compared based on the radiotherapy and surgical outcomes. Of these 200 patients, 49 had primary spinal tumors whereas 151 patients had spinal metastases. Patients underwent examination and physical pain assessment, which was based on a visual analog scale (VAS). In comparing multisession stereotactic radiosurgery versus radiation therapy for spinal metastasis, findings indicated similar pain control and quality of life post-operation. However, randomized, double-blinded treatment groups revealed that multisession stereotactic radiosurgery had slightly improved pain control based on a greater VAS score difference. Long-term follow-up studies can provide more information as to the long-term effects of these treatment options. In addition, tumor recurrence in response to the complications and adverse effects can be assessed with advanced future studies within the patient population [5].

Decompression surgery versus radiotherapy

A Cochrane Library systematic review by George et al. compared the use of decompression surgery, laminectomy, and corticosteroids, along with the use of radiation therapy [6]. Decompression surgery prior to radiation therapy appeared to have the most benefit compared to radiation therapy alone according to the Patchell study [7]; however, there was a significant bias in patients eligible for the trial as recruitment ended prematurely after 50% of their estimated sample size. The sample was also selected with a higher predilection for better outcomes with decompression surgery. As a result, 101 patients were enrolled and randomized in a clinical trial but not blinded to treatment. Funding for the Patchell study was provided by the National Cancer Institute and the National Institute for Neurological Disorders and Stroke.

While the International Stereotactic Radiosurgery Society (ISRS) guidelines use cord compression as an exclusion criterion for radiation therapy, the Patchell study required the presence of cord compression as an inclusion criterion [7]. Patients excluded from the study had any of the following features: the presence of more than one solitary lesion, paraplegia persisting more than 48 hours, and survival rate less than three months. The trial measured outcomes in regaining the ability to walk, pain control, mean time of survival, urinary continence, and lack of adverse effects. In each of the five outcomes, there was a statistically significant benefit in decompression surgery before radiation therapy over radiation alone. Eighty-four percent of patients in the combined treatment group regained the ability to walk compared to 57% receiving therapy alone. However, it did not increase the crude survival rate. Thus, treatment remains palliative while increasing the quality of life.

Further analysis found that the benefits of combined surgery and radiation therapy were restricted to those under 65 years of age, likely due to decreased complication rates, decreased rate of compression fractures, and better pre-diagnosis mobility [6]. Comorbidities also need to be considered before entering treatment, as comorbidities are a contraindication to aggressive treatment.

The urgency in which therapy needs to begin was not assessed; each patient underwent urgent decompression surgery upon enrolling in the trial. For maximum benefit, radiation treatment needed to begin within two weeks of the surgery. A small group of patients in the control arm deteriorated rapidly and were treated with decompression surgery after radiation therapy; this group had a 40% complication rate. This stresses the importance of decompression surgery prior to radiation therapy and provides patient-oriented evidence in the treatment plan of those with spinal metastases [6].

As many systematic reviews will suggest, the amount and quality of evidence are lacking due to strict selection criteria and the poor prognosis of spinal metastases. The Patchell study is considered to be a low Level of Evidence due to its selection bias and inherent flaws. Blinding was impractical in many studies due to the complexity of each treatment. The study was also halted immediately after the combination treatment group illustrated a clear benefit, further limiting the quality of data provided. The study also tended to find patients with histology resistant to radiation (renal cell carcinoma, melanoma), as those patients had a poor prognosis with radiation therapy alone. This reinforces the idea that in patients with malignancies sensitive to radiation and no signs of cord compression will benefit solely from radiation therapy. Alternatively, patients suffering from paraplegia would benefit from decompression surgery alone compared to radiation therapy, as 85% to 64% of patients regained function after each treatment [6].

Laminectomy combined with radiation therapy was similarly mentioned in the systematic review, although these older studies were not able to show a significant benefit in combination therapy compared to radiosurgery alone. The trial mentioned included only 29 patients and allocated those with complete myelographic block specifically to the surgery group, nullifying any randomization [6].

In summary, patients with spinal metastases from any non-central nervous system primary source may benefit from a higher quality of life with combination therapy. The researchers recommend decompression surgery prior to radiation therapy specifically in groups that meet these criteria: younger than age 65 with few comorbidities, estimated survival time of more than three months, a limited number of metastatic lesions, and good mobility prior to a transient loss of ambulation. In patients with metastases highly sensitive to radiation, radiotherapy alone is recommended unless there is significant cord compression hindering the patient's mobility, in which decompression surgery may be necessary [6].

A systematic review by Husain et al. found a limited number of articles available for spinal bone metastasis cases undergoing stereotactic surgery for the first time [8]. The review collected articles from 1946 to 2015 from PubMed, Embase, and the Cochrane Library. Four of the 14 studies focused on renal cell carcinoma metastasis, while one focused on breast cancer metastases. Only two were prospective studies funded by MD Anderson Cancer Center, while the remainder were retrospective. The total number of lesions treated between the 14 studies was 1,024. The purpose of each study was to report the outcome of stereotactic treatment. Blinding was not seen in the prospective studies due to the complexity of radiation treatment and the urgency of the patient’s condition. Local tumor control was reported in each study, although the definition of local control differed between them. The rate of local control averaged from 85% to 90%, while overall survival ranged from 15 to 19 months. There was a complete pain response in 54% compared to 23% in previous reviews; however, the study notes that there was no standard to what determined pain relief between the articles. Vertebral compression fractures were the most common adverse event, occurring in 9.4% of patients. The rate of adverse events was reduced with multifractionated dosing instead of single, high-dose radiation, with a 6% rate of adverse events versus 22% in single dosing. Adverse events were more likely in patients with increasing spinal instability and body mass index [8].

There was an inherent selection bias in the studies in that the patients were carefully evaluated on whether they qualified for radiation treatment. Current International Stereotactic Radiosurgery Society (ISRS) guidelines for patients that may benefit include patients with longer expected survival times due to oligometastases, patients with metastatic renal cell carcinoma, melanoma, sarcoma, and other sources typically resistant to radiation therapy, and patients with extension outside the bone to the paraspinal areas. Patients with limited benefit from radiation therapy include patients with survival time less than three months, patients with spinal mechanical instability, and patients with more than three metastatic lesions [8].

These guidelines have limited supporting evidence and are often based on expert opinion (Levels of Evidence IV and V). However, given the outcomes seen from each study in the review, it supports these guidelines and provides patient-oriented evidence that may change how oncologists and surgeons approach spinal metastases. Given the poor prognosis in these patients, it is difficult to properly stratify them into groups to remove confounding variables. The review notes that the primary malignancy was often different in histology compared to other studies and thus could have different sensitivities to treatment. However, a general idea of which patients would benefit from radiation therapy can be gained from summing the results of the articles. Overall, there is only one exclusion criterion with a high Level of Evidence - one based on the presence of neurological symptoms due to spinal cord compression or cauda equina syndrome. The next review provides supporting evidence that surgical decompression is beneficial (Level I) [8].

Klimo et al. conducted a meta-analysis in which they compared the outcomes of treating metastatic spinal disease with radiotherapy versus surgical decompression [8]. The primary outcome evaluated was ambulation, while secondary outcomes included pain control, sphincter function, survival, and post-treatment complications. Ambulation was analyzed in two ways: the proportion of patients that maintained their ambulatory function and the proportion who regained their ambulatory function. The researchers analyzed 24 studies that met their criteria of a clinical study in which adults with symptomatic metastatic spinal disease were treated with surgery (with or without postoperative adjunctive radiation) or radiation therapy alone. No exclusion criteria were listed. All but one of the studies were uncontrolled, nonrandomized prospective or retrospective cohort studies, with a total of 999 patients and 1,020 treated spinal lesions [9].

The researchers found that surgical patients had a 1.3 times greater chance of maintaining ambulatory function than those treated with radiation alone (p < 0.001). In addition, patients who were non-ambulatory preoperatively were twice as likely to regain their ambulatory function following surgical decompression than radiotherapy alone (p < 0.001). Pain was improved in an average of 90% of patients (range: 71% - 100%) who underwent surgical resection of their spinal metastasis, compared to 70% (range: 54% - 83%) who received radiation alone. Sphincter function was restored in 66% of surgical patients and only 26% of radiation-treated patients. The average one-year survival rates for surgical patients was 41%, while it was 24% in radiation-treated patients. However, the researchers acknowledged that the most significant factor that determined post-treatment survival was the primary histology of the spinal metastases. No significant radiotherapy complications were reported by the literature, while surgical complications occurred in 23% of cases and included wound infections, hardware migration, deep vein thrombosis/pulmonary embolisms, and new-onset neurologic deficits. Mortality within 30 days of the operation occurred in 6.3% of cases [9].

The researchers acknowledge that the lack of randomized and blinded trials limited their ability to draw clear correlations from their results. In addition, they were unable to limit studies to those in which patients were treated with surgical intervention alone in order to minimize any confounding effects that adjunctive radiation therapy would have had on the patient outcomes. However, the researchers acknowledged this limitation and were still able to find strong agreement among the studies supporting the improved outcomes of surgical intervention with or without adjunctive radiotherapy over radiation therapy alone. Given their stringent inclusion criteria, the researchers were successfully able to stratify and compare the desired outcomes among the studies and demonstrate consensus in their findings. Overall, the researchers reasoned that surgery, despite its potential complications, has comparable or even superior outcomes to radiation therapy in terms of improved function and survival of patients with spinal metastasis. The researchers concluded that all patients should be evaluated for possible surgical intervention [9].

Surgical long-term outcomes

Bakar et al. conducted a systematic review in which they evaluated positive preoperative predictors for improved outcomes following decompression with surgery for spinal metastases [10]. Specifically, the primary outcome evaluated was life expectancy, while secondary outcomes included ambulation, motor function, and neurologic function following radical surgical resection. The researchers analyzed 36 clinical studies, including eight prospective studies and 28 retrospective studies, that reported outcomes of surgery for spinal metastases. Animal, in vitro, and biomechanical studies, as well as book chapters and case reports, were excluded from their review. The study sizes ranged from 21 to 711 patients. The review found that predictors of improved survival following surgery included preoperative Karnofsky Performance Status (KPS), lack of visceral metastasis, preoperative ambulation, and decreased time since developing motor deficits. The KPS is a tool that assesses functional impairment based on the ability one is able to carry out without assistance in their daily living. A KPS ≥ 80% had a median survival of 13 months compared to patients with KPS < 40% with a median survival of two months. Several studies in the review found that the positive predictors of postoperative ambulatory status and motor function were the lack of visceral metastases, lumbar vertebrae involvement, preoperative performance status, and preoperative ambulation. Positive predictors of postoperative neurological function were decreased time since the onset of neurologic deficits. The review also found that of those who underwent surgical decompression followed by radiotherapy, 68% had improved sphincter function, 82% of paraparetic patients regained ambulation, and 88% had a resolution of their pain, compared to 33%, 64%, and 72% treated with radiation alone, respectively [10].

The systematic review excels in the number of studies it included and the strength of agreement among the studies. Many of them evaluated the surgical success based on similar preoperative predictors and outcomes, allowing for easy comparison. In addition, the results were directly relevant to the clinical application and patient care as they were centered on patient outcomes. However, a weakness in this systematic review is that it included a too broad array of studies, some of which where the surgical management was confounded by other treatment factors. For examples, several studies in the review included patients who were treated with surgical decompression followed by radiotherapy, confounding the effect these prognostic factors have surgical intervention alone. Nevertheless, the strong consensus among the majority of the studies highlights the overall significance of surgery. These results expand the candidate pool for patients who would potentially benefit from surgical resection. This study emphasizes the expanded role of surgery in treating patients with metastatic spinal disease, particularly when they possess the outlined positive predictors [10].

A systematic review by Clarke et al. found that the use of surgery in treating prostate cancer metastasis to the spine may benefit patients by reducing pain and preserving urinary continence [11]. The review selected articles from Ovid databases from their inception to October 2013. The basis of inclusion for these studies was spinal surgery for metastatic prostate cancer, whereas studies excluded patients who were either treated for all other tumors or if they were treated nonoperatively. Sixteen articles focused on overall survival, while six were added for nonsurvival questions of the review. Between the 16 studies focused on survival, 335 patients were enrolled, and a total of 553 patients were included for all 22 studies, with individual studies ranging from 8 to 114 patients [11].

The review assesses two main points of interest: survival rate, as well as overall pain and functional outcomes. With respect to survival, prostate-specific prognostic indicators were looked at, such as hormone-naivety, prostate-specific antigen (PSA), Gleason score, and KPS score. The review found that hormone naïve patients had an average of 55.4 months in comparison to hormone-refractory patients with an average survival of 6.4 months. KPS scores were also found to have a statistically significant difference in survival. Patients who had a KPS score ≥ 80 had a median survival of 22 months in comparison to 4.8 months in patients with a KPS score < 80. For functional outcomes, maintaining ambulation, urinary continence, and pain relief were the metrics used, and significant improvement was found in all three outcomes. Ninety-four percent of patients maintained strength or improved after one month, and 71% of patients were ambulatory, with 55% of preoperatively nonambulatory patients having ambulation restored. In terms of pain, 74% of patients had improvement one month after surgery with a decrease in median narcotic use and steroid use. With respect to urinary incontinence, 49% of patients regained continence. The mortality rate among all studies was 7% with a complication rate of 32%. Based off this data, the authors recommend spinal surgery for appropriately selected patients with prostatic metastases due to the overall improvement in the quality of life postoperatively, although there exists no marker that determines surgical consideration [11].

Overall, the review establishes a baseline for current data on the efficacy of prostatic metastases to the spine. As acknowledged in the review, current literature on the topic is limited, resulting in the relatively poor strength of data (Level IV) due to the small sample sizes in the studies reviewed. However, outcomes amongst all patients who underwent surgery were determined through examination of all patients included in this review. In terms of outcomes amongst all studies, there appears to be a significant benefit in terms of outcomes for patients with prostatic metastases, with clinically significant increases in motor strength and ambulation, regaining of urinary continence, and decrease in pain. Although the study states that it has yet to be seen how surgical techniques evolve in the field and how they affect outcomes, surgery appears to be an effective treatment option that may improve quality of life for patients [11].

Radiotherapy in the role of palliative care

A systematic review by Lutz et al. looked into updating current guidelines for palliative radiation therapy for bone metastases [12]. The previous set of guidelines were based around eight key questions proposed by the American Society for Radiology Oncology concerning the use of radiotherapies and implications for practice, such as long-term risks, indications for palliative radiation, and effective fractionation schemes. Updates to the current guidelines used the same eight key questions as the previous guidelines.

The authors searched PubMed records from 2009 to 2015 using keywords from the eight key questions. The review included 56 randomized controlled trials, meta-analyses, and prospective studies, and evidence was graded for overall guideline recommendations. Recommendations on guideline improvements were evidence-based answers to the key questions. Each recommendation was then graded based on an agreement between an expert panel, the strength of the recommendation itself, and the strength of the evidence supporting the recommendation [12].

Recommendations made by the panel found that radiation therapy is effective in pain relief, with 70 - 80% of patients undergoing therapy reporting a decrease in pain on a zero to 10 point scale in randomized controlled trials. In particular, for patients with limited life expectancy, a larger, single dose of radiation may be advisable, as a single 8 Gy fraction provides the same level of pain relief in a longer course of radiation therapy. Pain response differed by 8% between single and multiple fractions and was not significant. There exists no evidence that long-term adverse events affecting the lung or gastrointestinal system should preclude the use of single-fraction therapy. There was no demonstrable difference in toxicity, although there is an increase of pathologic fractures with single fraction at 3.3% incidence versus multiple fractions at 3%. For patients with persistent pain after one month of radiation therapy, it is recommended that they receive another dose of radiation. Fifty-eight percent of those who were irradiated demonstrated pain relief. Although therapy may be useful as a primary treatment, there is insufficient data to support this. Lastly, alternative therapies for painful metastases, such as surgery, radionuclides, bisphosphonates, or kyphoplasty, do not preclude the need for radiation therapy currently. Consequently, the authors recommend an interdisciplinary approach utilizing radiation therapy in conjunction with the alternative therapies [12].

The studies selected for review by Lutz et al. are large meta-analyses or randomized control trials that lend strong support to the survey of panelists and recommendations made [12]. The studies, however, focus on pain management generally only for one month after treatment, which limits evidence of long-term benefits of radiation therapy in comparison to surgical, radiopharmaceutical, or other methods of treatment. The review also acknowledges that there remains some ambiguity with defining “uncomplicated” bone metastases. The panel for agreement on recommendations also only consisted of members of the work group responsible for authoring the paper rather than a larger survey of radiation oncologists, which may predispose some voting on the guidelines to some bias. However, given the incredibly strong presentation of data, the conclusions and recommendations made based on the data affirm the utility of radiation therapy for palliative care of painful bone metastases. Their findings indicate an excellent reduction of pain for patients, as well as the use of single fraction radiation therapy versus multiple-fraction radiation therapy [12].

## Conclusions

Radiotherapy has remained the primary treatment modality in managing metastatic spinal disease for the past three decades. Given its non-invasive nature, growing precision with advanced technology, and relatively rare adverse effects, radiotherapy will continue to play a large role for the foreseeable future. The current standard for the management of bone metastases via radiation therapy has been well-established due to the efficacy of treatment, pain relief, and palliative care.

However, growing advances in surgical interventions have begun to challenge this current paradigm. As techniques become established and widely practiced, surgery has proven to be a viable therapeutic option that can provide patients with comparable, if not superior, outcomes to radiation therapy in terms of functional improvement, symptom resolution, and life expectancy extension. Given the proper symptomatic presentation, risk factors, and functional status, it is in the benefit of all patients with newly diagnosed metastatic spine disease that they be carefully evaluated for surgery as a potential primary treatment method.
